# Digital Mental Health and COVID-19: Using Technology Today to Accelerate the Curve on Access and Quality Tomorrow

**DOI:** 10.2196/18848

**Published:** 2020-03-26

**Authors:** John Torous, Keris Jän Myrick, Natali Rauseo-Ricupero, Joseph Firth

**Affiliations:** 1 Division of Digital Psychiatry Beth Israel Deaconess Medical Center Harvard Medical School Boston, MA United States; 2 Los Angeles County Department of Mental Health Los Angeles, CA United States; 3 Division of Psychology and Mental Health University of Manchester Manchester United Kingdom

**Keywords:** digital health, emergency response, telehealth, apps

## Abstract

As interest in and use of telehealth during the COVID-19 global pandemic increase, the potential of digital health to increase access and quality of mental health is becoming clear. Although the world today must “flatten the curve” of spread of the virus, we argue that now is the time to “accelerate and bend the curve” on digital health. Increased investments in digital health today will yield unprecedented access to high-quality mental health care. Focusing on personal experiences and projects from our diverse authorship team, we share selected examples of digital health innovations while acknowledging that no single piece can discuss all the impressive global efforts past and present. Exploring the success of telehealth during the present crisis and how technologies like apps can soon play a larger role, we discuss the need for workforce training, high-quality evidence, and digital equity among other factors critical for bending the curve further.

The COVID-19 crisis and global pandemic has highlighted the role of telehealth and digital tools like apps to offer care in times of need. Many clinicians and patients alike are now realizing the full potential of these digital tools, as they are forced to, for the first time, utilize them to connect in a time when in-person and face-to-face visits are impossible. Harnessing this surge in interest, enthusiasm, and acceptance has immediately been recognized as an opportunity for the field [1]. Thus, the field’s next steps will also be critical in ensuring digital health is used today to deliver the best care during the current crisis, ready for any resulting mental health spike following the immediate crisis, and prepared to support future crises as well as care as usual. In this perspective piece, we draw largely from our team’s experience with digital health and recognize the impressive global innovation and research in this space that cannot be captured in any single piece [2-9].

Telehealth is the right solution to deliver mental health care in today’s crisis. The only established contraindication to telehealth is a patient not wishing to partake. The temporary waiving of numerous rules and regulations around telehealth by the US government on March 17, 2020, was unprecedented [10]. It was made possible because of the strong and clear evidence base for the efficacy of telehealth and decades of high-quality research [11]. Our Boston team is already using telehealth to see patients during this current crisis, and feedback from patients as well as colleagues who are just starting to use telehealth suggests that this may be the new normal for many.

Digital therapy programs that can offer courses of evidence-based therapies also have a role in the crisis, given their unique potential for scalability. However, issues of real-world engagement with these programs [12] and high risk of bias in many studies [13] warrant caution in ensuring that plans for encouraging and maintaining meaningful engagement are in place before purchasing these programs or services. Workflow integration issues are also critical to consider when beginning to utilize these types of programs in care settings, and lack of attention here can lead to low uptake and support by both patients and staff [14,15]. New innovations in augmented and virtual reality systems hold great promise [16], but are not yet easily scalable or accessible to all for use in this current crisis.

Tools like apps also have an important role, given their availability and scalability, but with similar caveats. Current evidence for apps for behavior change remains limited [17], and self-help for mental health remains equally limited [18]. Although companies will boast about positive outcomes from randomized controlled trials [19], results from higher-quality studies with valid comparisons groups, proper statistical analysis, and low risk of bias do not tell the same story [18,20]. This is not to say that mobile technologies do not have a role in care. Rather, they possess a tremendous and still largely untapped potential to augment and extend care. Our team in Boston uses mobile apps to better understand the unique lived experience of each patient [21] in both our research as well as our digital clinic. Using apps in conjunction with current care has been shown to greatly increase the efficacy of app interventions [18]; in addition, the sensor, survey, and digital phenotyping data can be used to make more informed, data-driven, and evidence-based decisions about care. Our (JT and NRR) experiences of running a novel “digital clinic” in Boston offering such hybrid care confirm the theoretical advantages with real-world benefits. Now, bringing app data into telehealth visits offers a practical means for patients to share data of their lived experience during this crisis (eg, changing exercise and stress levels or response to new medications) and practice new therapy skills between sessions.

In our experience, the most effective apps are the ones that can be customized to each patient and fit with their personal care goals and needs as well as apps for peer support. Our team is fortunate in that we have created our own app tool (freely available and open source) [22] that we often utilize in research and care, but we realize that different apps are often needed for different situations. Picking from those different apps is challenging as many offer little protection of user data, make exaggerated claims, may be ineffective, and often are quickly abandoned because of usability issues [18,23,24]. Thus, careful attention is warranted when recommending such apps to patients [25]. Thus, relying on static lists of “top apps” or others’ scoring systems for selecting apps is often unhelpful and even dangerous, given how out of date these recommendations are [26]. Although many app-evaluation tools exist, our research and development of the American Psychiatric Association’s app evaluation framework offers a practical and ready-to-use resource today for both patients and clinicians [27].

One underdeveloped area for digital therapy and mental health apps is the remote delivery of “lifestyle interventions.” There is now increasing evidence that lifestyle factors such as physical exercise, sleep, and healthy diet play an important role in self-management of mental health conditions [28]. Consideration of these lifestyle factors for mental health may be particularly important during periods of isolation/prolonged home time, due to the adverse psychological effects of reduced exercise [29] or prolonged sedentary behavior [30], and the ongoing debate about certain types of screen time and social media usage (with quality of online interactions mattering more than time) [31]. Social distancing (which is actually physical space distancing) and self-quarantine will place millions of people at higher risk of disruption to lifestyles that likely benefited their mental health. Nonetheless, digital technologies and smartphone apps may also present a novel platform for the remote delivery of lifestyle interventions [32]. However, there is still a great need for further research to establish how this can be done in an engaging and effective way, to reach those with mental illness.

Looking beyond the immediate consequences of infection with the virus and the mental health impact of self-quarantine and social distancing, a second mental health crisis looms. In times of economic recession, there is often high prevalence of mental health disorders, misuse of substances (or substance use disorder), and deaths from suicide [33]. The need for more mental health services will tax an already overburdened health care system, and digital solutions will be called upon again. Learning from decades of prior research and experience, hybrid solutions that offer a blend of face-to-face and online or app-based treatment will be the most effective solution [14,18].

Ensuring the field advances from the recent interest and use in digital health to further accelerate access and quality of care beyond these immediate and imminent crises is the next challenge. The efforts reducing implementation barriers to video visits (also known as synchronous telehealth) during this current crisis highlight the potential to bend the curve on access to care (Figure 1). Further efforts and investments will be required to now have more access and quality as the field aims to fully utilize technologies like apps and beyond. Investing in the evidence, outcomes, workforce, engagement, and ethical uses of these newer technologies and innovations will allow them to bend the curve and truly deliver on their full potential, just as we are seeing telehealth today benefiting from its legacy of prior investments.

**Figure 1:**
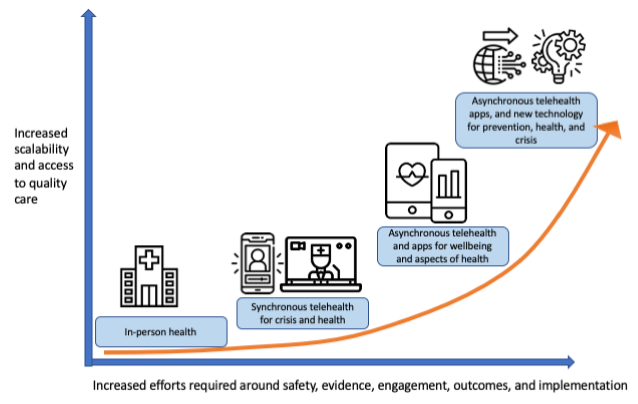
Bending the curve further on access and quality of care will require increased efforts around safety, evidence, engagement, outcomes, and implementation. However, these increased efforts will yield greater returns at each step. The COVID-19 crisis has (at least temporarily) removed implementation barriers to synchronous telehealth through regulatory changes, and the evidence, safety, and engagement were already in place before. The next steps to use apps toward asynchronous telehealth will require continued effort but yield even greater increases in access to high-quality care.

Among some of these new efforts required, a critical one is teaching medical professionals, trainees, and peer support specialists how to use digital and mobile technologies for delivering care. Frameworks for competencies already exist [34,35], and a few have already been implemented. Our personal experience in teaching psychiatry residents about mobile mental health in formal didactics has been positive, and there are already many examples of teaching telehealth [36,37]. Training new care team members around digital health, a role we have termed a digital navigator [38], offers opportunities to easily liaison between digital and classical care. Although training does not offer an immediate solution to the current crisis, it creates the workforce and builds the capacity to support increased access to care for the mental health sequelae of the current crisis and readiness for the next.

Training new providers is, however, only half the picture. Ensuring all patients, especially the most vulnerable ones, have the digital literacy and competency to partake in digital care is a matter of equity and social justice. Many people today find it easy to use their smartphone to set reminders, download apps, join video calls, and connect with peers. However, many people do not, and offering training and skills building is critical to ensure digital health actually offers help to those who need it the most [39]. Our Digital Opportunities for Outcomes in Recovery Services (DOORS) program offers 6-8 weeks of group sessions to develop smartphone skills and competences that have been well received by those with serious mental illness [38] and is freely shareable for others to expand upon. Focusing on and developing programs like this one, which ensures everyone is able to connect and receive care, may not have the attention-grabbing status like artificial intelligence and virtual reality, but such programs are likely of more importance now more than ever.

We must also be aware of the disparities that impact people with low income, those receiving public benefits, and cultural and linguistically diverse communities that may not have access to even basic technology including digital mobile technology. Subsidized phone programs such as Lifeline Assistance may have data, speed, and calling limits. As such, the people we serve may have to make choices about what they will download and how they will use their mobile devices, even though greater opportunities exist. Further, we need to understand how people use their mobile devices—from sharing them with family, housemates, and friends to “renting them for a day” to access funds to meet basic needs—as this will impact privacy as well as research if we are not sure whose hands the phone really is in. During this time of self-quarantine and stay-at-home orders, if one is homeless or unstably housed, charging the phone or laptop is a huge barrier as is finding public or library hotspots. For now, and in the future, we need to prepare the workforce to conduct street psychiatry and outreach work in order to carry chargers and portable hotspots and provide treatment via street psychiatry or connecting with peers or outreach workers. Some people still have and use flip phones (non-smartphones), and this population cannot be ignored. For digital mental health to impact those who are most vulnerable we must be vigilant when addressing these disparities. Ensuring digital data collected for mental health purposes is not repurposed and used for surveillance or sold for profits/marketing is critical, as any lack of trust or transparency in such a system will erode meaningful use. A focus on equity and ethics will ensure digital health truly increases access to care [40].

The COVID-19 crisis and global pandemic may be the defining moment for digital mental health, but what that definition will be remains unknown. Ensuring the right use of telehealth and app tools today in this crisis and investment in people and training to support them tomorrow during the potential mental health fallout of the current crisis as well as readiness for tomorrow can cement the future of digital mental health as simply mental health. Bending the curve in the right direction (Figure 1) will require funding, research, policy changes, training, and equity, but these investments will continue to yield higher returns at every step.

## Abbreviations

DOORS: Digital Opportunities for Outcomes in Recovery Services
